# Development of innovative tripartite partnership for China’s engagement in global health: recommendations from China-Tanzania Cooperation Project on Malaria Control

**DOI:** 10.1186/s40249-024-01178-4

**Published:** 2024-03-05

**Authors:** Xuejiao Ma, Shenning Lu, Wei Ding, Shanying Deng, Duoquan Wang, Ning Xiao, Yeromin Mlacha, Lewis Husain, Xiaonong Zhou

**Affiliations:** 1 National Institute of Parasitic Diseases, Chinese Center for Disease Control and Prevention (Chinese Center for Tropical Diseases Research), NHC Key Laboratory of Parasite and Vector Biology, WHO Collaborating Center for Tropical Diseases, National Center for International Research On Tropical Diseases, Shanghai, China; 2 University of Hong Kong, Hong Kong, China; 3 School of Global Health, Chinese Center for Tropical Diseases Research, Shanghai Jiao Tong University School of Medicine, Shanghai, China; 4 Ifakara Health Institute, Dar-es-Salaam, Tanzania; 5 Institute of Development Studies, Brighton, UK

## Background

Tripartite partnership, compared with the bilateral model, is envisioned as an opportunity to foster stronger and more trusting partnerships [1]. However, China has historically been characterized as principally reliant on bilateral engagement in global health [2]. The China-Tanzania Cooperation Project on Malaria Control was conducted by National Institute of Parasitic Diseases at China CDC and Chinese Center for Tropical Diseases Research, Ifakara Health Institute in Tanzania and funding agencies from 2015 to 2022, with an aim to explore the applicability of Chinese experience on malaria control to reduce malaria disease burden in the local context of Tanzania. In the tripartite project funded by the former UK Department for International Development (DFID) and later the Bill & Melinda Gates Foundation (BMGF), China’s partners were expected to share the extensively parallel practices achieved from the control and elimination of malaria for over 70 years, UK’s partners were to deliver resources and design global health strategies, while Tanzanian local staff collaborated with triangular partners to ensure the project implementation [3]. In the project, the trilateral cooperation has transparent management mechanism to ensure the concrete management and implementation of the project [4] (Additional file 1: Figure S1). This Brief is to provide recommendations for China’s engagement in global health by distilling the experience from the innovative tripartite partnership of the project.

## Achievements of the project tripartite partnership

Impressive achievements were made through the pioneering efforts of triangular stakeholders of the project: (1) 1,7-malaria Reactive Community-based Testing and Response (1,7-mRCTR) was successfully derived from local context sharing Chinese 1-3-7 norm through the field implementation; (2) malaria prevalence in the intervention wards declined by 81% from 2015 to 2018 and further declined by 55% from 2018 to 2022 [3]; (3) a local team composed of 37 Tanzanian community health workers were built to be paired with Chinese on-site technical staff to ensure the project implementation and sustainability[3]; (4) the project’s effectiveness and achievements contributed to the scaling up of 1,7-mRCTR in three other African countries including Burkina Faso, Senegal, and Zambia for sustainable development [4].

## Challenges and recommendations

The project also encountered some challenges in this trilateral partnership, (1) China’s immature systems for global health engagement with scarce resources allocated to this innovative partnership [5], e.g. the complicated procedures of funding transfer from China to Tanzania, the time-consuming visa application for Chinese staff’s field support, and the scarce resources. Therefore, it is essential to mobilize resources for the trilateral collaboration with developing a solid system for China’s participation in global health. In order to effectively mobilize resources for these trilateral collaborations, it is imperative to develop a robust logistics system that underpins China's active participation in global health endeavors. This would necessitate the optimization of domestic structures and the introduction of supportive policies aimed at empowering the Chinese workforce to contribute to global health. For instance, simplifying bureaucratic procedures such as visa applications and the transfer of funds is crucial to ensure that the field work is executed without unnecessary delay.

(2) Limited competences and experiences for China’s global health workforce, e.g. lack of field practices due to limited resources and opportunities to develop competences to coordinate multiple stakeholders and ensure coherence across sectors after changing from bilateral assistance to multilateral global health cooperation [4]. It shall be necessary to strengthen global health competences by continuously enhancing the cross-cultural field practices through triangular cooperation**.** More opportunities to work abroad such as the triangular cooperation projects should be provided to the Chinese workforce in global health so they can gain practical knowledge in the field and become familiar with international regulations in a cross-cultural setting. In the project, the paired learning by doing approach might be an alternative way for competence enhancement [4]. What’s more, it’s indispensable to work on site with local personnel and different stakeholders at the frontline in the global health arena.

## Conclusions

The innovative tripartite partnership may provide a model for China’s future engagement in global health cooperation. Scaling up tripartite partnerships for engagement in global health, China can leverage its strengths and technologies, while utilizing the advantages of other partners to address pressing global health challenges and promote sustainable development.

### Supplementary Information

**Additional file 1: Figure S1.** Tripartite partnership of China-Tanzania Cooperation Project on Malaria Control. *CN* China; *UK* United Kingdom; *BMGF* Bill& Melinda Gates Foundation; *TZ* Tanzania; *1,7-mRCTR* 1,7-malaria Reactive Community-based Testing and Response.

## Data Availability

All the data came from the references.

## Abbreviations

UK: United Kingdom
DFID: Department for International Development
BMGF: Bill & Melinda Gates Foundation
CDC: Center for Disease Control
1,7-mRCTR: 1,7-Malaria Reactive Community-based Testing and Response
