# Supplementary figures and images for: Development of innovative tripartite partnership for China’s engagement in global health: recommendations from China-Tanzania Cooperation Project on Malaria Control
Source: Infect Dis Poverty. 2024 Mar 5;13:22. doi: 10.1186/s40249-024-01178-4 (PMC10913682; doi:10.1186/s40249-024-01178-4)

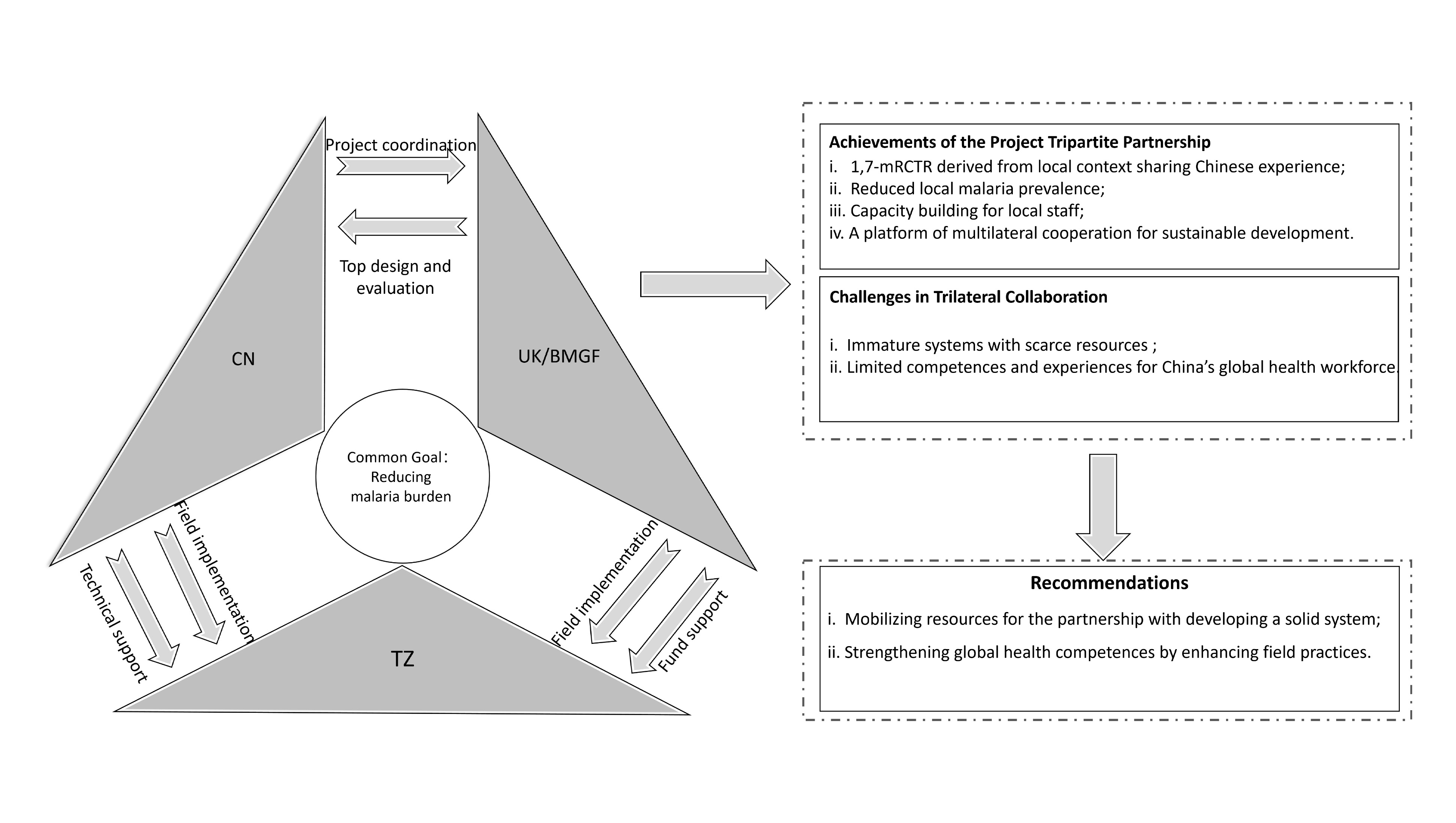

Supplement: Supplementary file 1 — Additional file 1: Figure S1. Tripartite partnership of China-Tanzania Cooperation Project on Malaria Control. CN China; UK United Kingdom; BMGF Bill& Melinda Gates Foundation; TZ Tanzania; 1,7-mRCTR 1,7-malaria Reactive Community-based Testing and Response. [file 40249_2024_1178_MOESM1_ESM.jpg]
